# Desmoid Tumor of the Porta Hepatis: A Rare Location With Unusual Clinical Presentation

**DOI:** 10.1155/crhe/1253334

**Published:** 2026-05-12

**Authors:** Adila Adilli, Altay Aliyev, Yıldırım Karslıoğlu, Gulen Bulbul Dogusoy, Eldar Ahmadov, Parvana Asgarova, Farida Agayarlı, Iqbal Babazade, Arturan Ibrahimli, Gunel Ziyadova

**Affiliations:** ^1^ Department of Pathology, Bona Dea International Hospital, Baku, Azerbaijan; ^2^ Department of Medical Oncology, Bona Dea International Hospital, Baku, Azerbaijan; ^3^ Department of Pathology, Memorial Pathology Laboratory, Istanbul, Turkey; ^4^ Department of General Surgery, Bona Dea International Hospital, Baku, Azerbaijan; ^5^ Department of Radiology, Bona Dea International Hospital, Baku, Azerbaijan; ^6^ Azerbaijan Medical University, Baku, Azerbaijan, amu.edu.az; ^7^ Department of Obstetrics and Gynecology, Bona Dea International Hospital, Baku, Azerbaijan

**Keywords:** abdominal wall, biopsy, desmoid tumor, fibromatosis, Gardner syndrome

## Abstract

**Background:**

Desmoid tumor is a locally aggressive fibroblastic/myofibroblastic neoplasm frequently arising in deep soft tissues. Although it can be well circumscribed, a desmoid tumor generally infiltrates surrounding tissues and tends to recur locally without metastasizing. While commonly arising in the extremities and abdominal wall, primary hepatic involvement is exceedingly uncommon.

**Case Report:**

We report the case of a 20‐year‐old woman presenting with progressive jaundice, pruritus, weight loss, and abdominal pain. Imaging revealed a 3 × 3 cm hepatic hilar mass with bile duct dilation, initially suggestive of cholangiocarcinoma. Histopathological examination following a tru‐cut biopsy demonstrated features consistent with desmoid‐type fibromatosis, confirmed by nuclear β‐catenin positivity. Due to clinical deterioration, the patient underwent a left hemihepatectomy. The postoperative course was uneventful. Adjuvant tamoxifen therapy was administered, and follow‐up imaging showed no recurrence. Despite the hormonal changes of a subsequent pregnancy, no disease progression was observed.

**Conclusion:**

This report highlights an extremely rare presentation of desmoid tumor in the hepatic hilum, mimicking malignancy. It underscores the importance of histopathological confirmation, multidisciplinary management, and individualized follow‐up strategies, especially in women of reproductive age.

## 1. Introduction

Desmoid tumors (DTs) are monoclonal, spindle cell proliferations arising from musculoaponeurotic structures [1]. Although histologically benign, these deep‐seated myofibroblastic neoplasms are notorious for their locally aggressive, infiltrative growth and high propensity for recurrence, despite lacking metastatic potential. Although Mueller in 1838 coined the term DT (derived from the Greek *desmos*, which means tendon‐like), the first description of the tumor is credited to McFarlane, who reported the disease occurring in the abdominal wall of a young woman after delivery in 1832 [2].

They have been classified according to anatomical location as extraabdominal, abdominal wall, and intraabdominal variants. Approximately 30%–40% of cases are diagnosed in the extremities, with lesions also emerging in the retroperitoneum or abdominal cavity (15%), abdominal wall (20%), and chest wall (10%–15%). Less common sites include the head and neck, paraspinal region, and flank [3]. Although the precise etiology remains unclear, contributing factors include local injury such as postoperative scars, hormonal influences (with estrogen playing a key role during the reproductive years, pregnancy, and its regression after menopause or with tamoxifen therapy), and genetic predispositions, notably adenomatous polyposis coli (APC) gene mutations associated with familial adenomatous polyposis (FAP) or Gardner’s syndrome [4–7].

Pregnancy‐related hormonal changes are known to influence DT growth. While some cases exhibit tumor progression during gestation, spontaneous regression postpartum has also been documented. Interestingly, our patient, despite having a history of pregnancy and childbirth, did not experience recurrence, further emphasizing the unpredictable nature of hormonal modulation in DT behavior. This observation is consistent with previous findings indicating that although estrogen can stimulate tumor growth, recurrence after pregnancy does not occur in all cases—especially when additional genetic predispositions, such as APC mutations, are absent [8].

The diagnosis is based on a histopathological examination that shows spindle cell proliferation without overt atypia and with nuclear beta‐catenin positivity in most of the cases. While surgical excision with clear margins is the primary treatment modality, the unpredictable biology and occasional occurrence in exceptional anatomical locations necessitate innovative therapeutic strategies.

Hepatic DTs are extremely rare. There has been only one case in the literature describing a hepatic mass, but in that case, the lesion is located in the diaphragmatic area [1]. In the present report, we describe a 20‐year‐old female who presented with weight loss, pruritus, jaundice, and abdominal pain. Radiologic evaluation revealed a 3 × 3 cm mass in the hepatic hilum with associated intrahepatic bile duct dilation, initially suggestive of cholangiocarcinoma. However, a core needle biopsy biopsy and subsequent beta‐catenin immunohistochemistry confirmed the diagnosis of a DT. This represents, to the best of our knowledge, the first reported case of a DT arising in the hepatic perihilar region mimicking cholangiocarcinoma, a finding that contrasts with a previously reported aggressive DT in the gallbladder fossa that did not involve the porta hepatis [9].

## 2. Case Presentation

**FIGURE 1:**
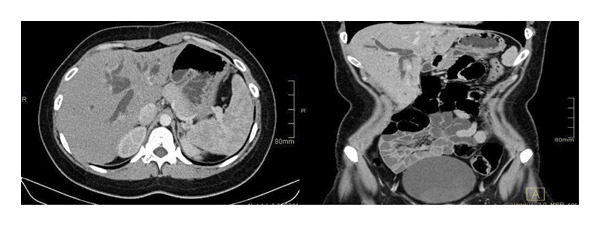
Axial venous phase CT: at the hepatic hilum (central bile duct level), an iso to hypodense, hypovascular mass lesion is seen, causing bile duct dilation, barely distinguishable from the surrounding parenchyma.

This case involves a 20‐year‐old Caucasian woman who presented to our hospital’s general surgery department with complaints of weight loss, pruritus, jaundice, and abdominal pain persisting for three months. Initial laboratory tests revealed significantly elevated total bilirubin (359 μmol/L; reference range: 5.1–20.5 μmol/L), direct bilirubin (256 μmol/L; reference range: 0–5.1 μmol/L), and indirect bilirubin (102 μmol/L; reference range: 3.4–17.1 μmol/L), which clearly indicated a biliary origin for the patient’s pathophysiology and were directly correlated with her clinical symptoms. An abdominal computed tomography (CT) scan performed on March 31, 2022, revealed a well‐defined 3 × 3 cm mass at the hepatic hilum, causing intrahepatic bile duct dilation and extending into the lumen. No significant periportal lymphadenopathy was noted (Figure 1). With these imaging studies, a preliminary diagnosis was made as hilar cholangiocarcinoma (Klatskin tumor), and a percutaneous tru‐cut biopsy on April 2, 2022, was performed.

**FIGURE 2:**
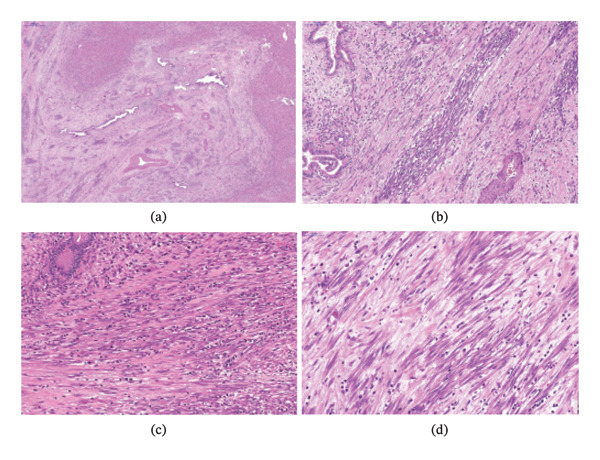
(a) Low‐power histological view showing portal hepatis surrounded by a fibroblastic appearing spindle cell (hematoxylin and eosin; magnification, × 100). (b), (c), (d) High‐power histological view showing proliferation of uniform slender spindle‐shaped cells arranged in bundles and fascicles, which are surrounded by varying amounts of collagen (hematoxylin and eosin; magnification: × 200, × 400).

**FIGURE 3:**
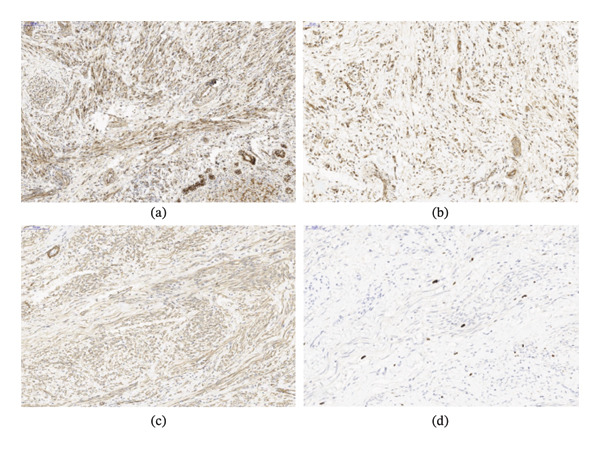
(a), (b) Beta‐catenin immunohistochemical stain diffusely highlighting the lesional cells in a cytoplasmic pattern with scattered nuclear reactivity, typical of DTF (IHC × 100, × 200). (c) Immunohistochemical stain for smooth muscle actin with positive staining, confirming the myofibroblastic nature of the tumor cells (IHC × 200). (d) Low Ki‐67 positivity in fibromatous proliferation (IHC × 200).

Microscopically, the tissue samples showed a mesenchymal lesion highly suggestive of fibromatosis characterized by proliferation of uniform slender spindle‐shaped cells arranged in bundles and fascicles, which are surrounded by varying amounts of collagen (Figure 2). There was no evidence of nuclear pleomorphism, necrosis, or increased mitotic activity. Immunohistochemical analysis demonstrated positive against β‐catenin (nuclear expression), SMA, desmin, Cyclin D1, and caldesmon, and negative for Myo‐D1, DOG‐1, calretinin, STAT6, MDM2, CD34, pancytokeratin, and ALK1 (Figure 3). The diagnosis was made as “low‐grade spindle cell mesenchymal tumor” with a comment stating the possibility of DT.

**FIGURE 4:**
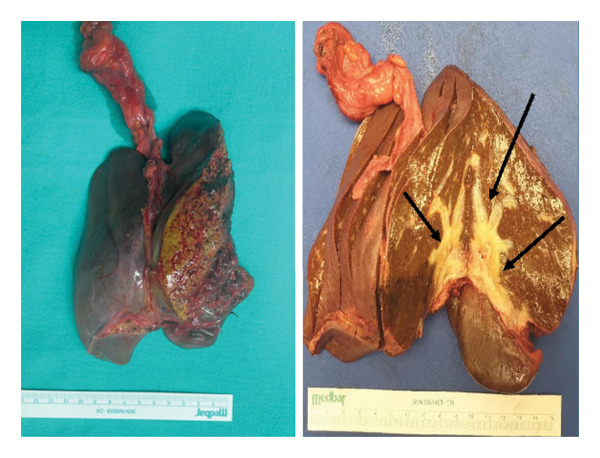
Macroscopically, desmoid tumors were grayish‐white, scar‐like masses located at the hepatic hilus, partially extending to the right and left lobes, and surrounding the hilar structures (arrows).

As the tumor had invaded predominantly toward the left lobe, causing progressive obstructive jaundice, persistent abdominal pain, and markedly elevated bilirubin levels, a left hemihepatectomy was performed on April 20, 2022, 18 days after the biopsy. Given the involvement of both the right and left hepatic ducts by the hilar tumor, biliary reconstruction was required; a right‐sided Roux‐en‐Y hepaticojejunostomy was performed to restore biliary drainage. On macroscopic examination, a grayish‐white, scar‐like mass measuring 4 × 3.2 × 2 cm located at the hepatic hilus, partially extending to the right and left lobes, and surrounding the hilar structures was observed (Figure 4). The tumor was sampled and submitted totally for histological examination. Histopathological assessment of the resected tumor confirmed the features observed in the preoperative biopsy, further supporting the diagnosis of desmoid‐type fibromatosis (DF). Subsequent colonoscopy and endoscopy performed on October 29, 2022, did not reveal any colorectal polyps, effectively ruling out FAP.

Two months after surgery, the patient was started on tamoxifen 20 mg. Since she remained stable and had no contraindications from the gynecological evaluation, the tamoxifen dose was increased to 40 mg. However, due to nausea and diarrhea 3 months later, the dose was reduced back to 20 mg. Given the patient’s young age, she was monitored every 3–6 months while on tamoxifen. In consideration of her family planning and desire for pregnancy, tamoxifen was discontinued 3 months before her planned conception.

Fourteen months after the surgery (on June 25, 2023), the patient became pregnant as planned, having started a family and wanting children, and subsequently gave birth to a healthy baby on February 17, 2024. An ultrasound performed at 27 weeks of gestation confirmed a normal intrauterine pregnancy.

Given the established role of estrogen in the pathophysiology of DTs, the patient was closely monitored throughout her pregnancy. Despite the expected physiological increase in estrogen levels in the third trimester, no significant tumor recurrence or progression was observed in the postpartum period. The MR image obtained approximately 2 months before delivery, approximately 20 months after DT resection, also showed no recurrence.

This case highlights a rare presentation of primary hepatic DT and its multidisciplinary management. It underscores the role of surgical and hormonal therapy in the treatment of DTs and emphasizes the importance of careful monitoring during pregnancy.

## 3. Discussion

DT is a rare lesion representing < 3% of all soft tissue tumors with an estimated incidence of 2‐4 new cases per million per year [10]. Generally, individuals in the age group of 15–60 years can be affected. DTs are rare in the young and in the elderly, and more common in women than in men [2, 11, 12]. Also known as aggressive fibromatosis or DF, DTs are local aggressive tumors which can develop anywhere in the body. They invade surrounding tissues and, in some cases, can be very difficult to keep under control because of their tendency to recur [13]. The WHO classifies DTs as intermediate (locally aggressive, nonmetastasizing) fibroblastic and myofibroblastic tumors [3].

There are two main types of DTs: sporadic tumors, which account for the majority (85%–90%) and are associated with somatic mutations in the β‐catenin (CTNNB1) gene, and those linked to APC gene mutations. The latter, making up 10%–15% of cases, are often seen in individuals with FAP, a genetic condition that increases tumor risk [9, 13, 14]. Sporadic DTs, which make up the majority of cases, are influenced by factors beyond genetic mutations. Trauma, including prior surgical procedures or mechanical stress, has been linked to their development [15, 16]. In addition, hormonal influences, particularly estrogen, play a key role in tumor growth. The increased incidence of DF during and shortly after pregnancy, as well as reports of spontaneous regression and disease stabilization with hormonal therapies, further support the role of estrogens in its pathogenesis and progression [16].

Following traumatic injury, the body’s wound healing process may contribute to the development of DTs. During tissue repair, fibroblasts become activated and generate fibrotic tissue to facilitate healing. Under normal circumstances, fibroblasts return to a quiescent state once the wound has healed. However, in certain cases, this regulatory process becomes disrupted, leading to uncontrolled fibroblastic activity, excessive fibrosis, and ultimately, DT formation. Fibroblast activation is largely regulated by the Wnt‐ and Notch‐signaling pathways [8, 17–19]. In DTs, however, a key characteristic of fibroblasts is a disrupted Wnt pathway, primarily due to gain‐of‐function mutations in Exon 3 of the CTNNB1 gene. The CTNNB1 gene codes for β‐catenin, a protein that plays a crucial role in regulating cell activity. Normally, once wound healing is complete, β‐catenin is broken down by proteasomes, causing fibroblasts to return to a resting state. However, the gain‐of‐function mutation alters the structure of β‐catenin, preventing its degradation and keeping the fibroblasts in a constantly active state. This persistent activation can also lead to the formation of keloids, a phenomenon observed in several of our patients.

They have been classified according to anatomical location as extraabdominal, abdominal wall, and intraabdominal. Intraabdominal DTs are rare, with the majority occurring in the mesentery or retroperitoneum [20]. However, DTs in solid organs such as the pancreas have been reported in isolated cases, including a recent study describing a pancreatic DT [13]. In addition, a rare case of aggressive intraabdominal DF following cholecystectomy was reported in a 67‐year‐old male, where the tumor presented in the gallbladder fossa, demonstrating complications such as gastric outlet and biliary obstruction, portal vein narrowing, and encasement of the hepatic artery [9].

To our knowledge, only one case of a primary hepatic DT has been reported in the literature. Our case thus represents the second reported instance, specifically located in the perihilar region of the liver. Notably, colonoscopy performed to exclude FAP revealed no alterations in the colonic mucosa. Furthermore, while trauma or surgical history is considered a risk factor for the development of DTs, our patient had no prior history of abdominal trauma or surgery.

DTs typically present as a slow‐growing mass, with symptoms varying based on their location. Intraabdominal DTs may remain asymptomatic; however, in some cases, they can lead to complications such as intestinal obstruction, vascular compression, urinary tract obstruction, or nerve involvement [21, 22]. In our case, the DT was located in the hepatic perihilar region, and the patient presented with symptoms including weight loss, pruritus, jaundice, and abdominal pain. Diagnosing a DT based solely on radiological imaging and laboratory findings is extremely challenging in such cases. In our patient, imaging results strongly suggested hepatic hilar cholangiocarcinoma (Klatskin tumor), leading to the decision to perform a tru‐cut biopsy for definitive diagnosis.

Intraabdominal DTs are mesenchymal neoplasms that lack organ specificity and can arise in various locations within the abdominal cavity. Therefore, distinguishing them from other tumors such as GIST, sarcoma, lymphoma, and neurogenic tumors is crucial. [23]. A histological assessment is essential for confirming the diagnosis. While an incisional biopsy is the preferred method, a carefully performed biopsy may also provide sufficient information [24]. In our case, a biopsy was performed first, and we diagnosed it as a “low‐grade spindle cell mesenchymal tumor” with a comment stating the possibility of a DT.

DTs present a range of treatment options, and their management often requires a case‐by‐case approach. In our patient, surgical resection was deemed both necessary and feasible, leading to surgery 18 days after the initial biopsy. Histopathological analysis of the left hemihepatectomy specimen confirmed the diagnosis of a DT. A focal area of positive surgical margin was observed histologically. Although a complete macroscopic resection was achieved, the presence of focal margin positivity is recognized as a significant risk factor for local recurrence [25].

Currently, there is no standardized guideline for postoperative surveillance of DTs. Given the reported recurrence rate of up to 85% following surgical excision, surgery should be reserved for cases where it is absolutely essential [26].

Many reports suggest complementary treatment with tamoxifen, chemotherapy, or radiotherapy in DTs [27]. Management varies based on tumor location and progression, with options including active surveillance, NSAIDs, hormonal therapy (e.g., tamoxifen), targeted therapy, and systemic chemotherapy [28, 29].

It has also been reported that estrogen might be involved in the development and growth of DTs; hence, they often occur in women, particularly in multiparous women. Estrogen receptors are highly expressed in a large number of DTs, and tamoxifen has recently been reported to be effective as a treatment [30].

Several cases of complete tumor regression have been reported following tamoxifen treatment for DTs. One such case involved a 47‐year‐old man who developed a recurrent 8‐cm abdominal mass after surgery. Despite its proximity to vital structures, complete resection was not possible. However, after 3 years of tamoxifen treatment, the tumor fully regressed, with no recurrence in the following 3 years. This case demonstrates that even large DTs can be successfully treated with tamoxifen, regardless of estrogen receptor status [31].

In our case, tamoxifen therapy was chosen over high‐dose steroids or cytotoxic agents due to the patient’s young age and the relatively lower risk of adverse effects associated with tamoxifen. The patient was counseled about the increased risk of recurrence during pregnancy, but she was determined to proceed. Tamoxifen therapy was discontinued 3 months before the planned pregnancy. Notably, the patient successfully delivered a healthy baby in February 2024 and has remained recurrence‐free for the past two years, under regular six‐monthly CT follow‐ups.

## 4. Conclusion

In summary, this case represents a unique presentation of DF arising in the hepatic hilum, an anatomical site not commonly associated with this tumor. The mass mimicked a malignant process, emphasizing the diagnostic challenges it may pose. The patient’s excellent outcome following surgery and hormonal therapy, along with the absence of recurrence during pregnancy, supports a personalized and carefully monitored approach to management—particularly in young women. This case contributes valuable insight into the rare hepatic manifestation of DTs and highlights the need for awareness of such atypical presentations to avoid unnecessary aggressive interventions.

## Funding

No funding was received for this study.

## Ethics Statement

No institutional ethics committee approval was required for the publication of this single case report, as it involved routine clinical care and did not constitute a research study involving human subjects.

## Consent

No written consent has been obtained from the patients as there are no patient identifiable data included in this case report.

## Conflicts of Interest

The authors declare no conflicts of interest.

## Data Availability

The data that support the findings of this study are available from the corresponding author upon reasonable request.
